# Adjuvant Radiotherapy for Thymic Neuroendocrine Tumors: A Case Report and Review of the Literature

**DOI:** 10.7759/cureus.1115

**Published:** 2017-03-26

**Authors:** Omar Iskanderani, David Roberge, Geneviève Coulombe

**Affiliations:** 1 Department of Radiation Oncology, Centre Hospitalier de l'Université de Montréal (CHUM); 2 Department of Oncology, Division of Radiation Oncology, McGill University Health Center

**Keywords:** thymic neuroendocrine tumors, adjuvant radiotherapy, neuroendocrine tumors

## Abstract

Thymic carcinoid tumors are very rare. Between two and four percent of carcinoids originate from the thymus with an estimated incidence of 1.5 to 3 per 10,000,000 persons per year. Thymic carcinoids can be associated with the multiple endocrine neoplasia (MEN) type 1. The principal treatment is surgical resection. The potential roles of systemic and radiation treatments are a matter of debate. We describe the successful multidisciplinary treatment of a case of thymic carcinoid associated with MEN and review the literature pertaining to the use of adjuvant thoracic radiation.

## Introduction

Thymic carcinoid tumors are very rare. Between two and four percent of carcinoids originate from the thymus with an estimated incidence of 1.5 to 3 per 10,000,000 persons per year [1]. Thymic carcinoids, also referred to as thymic neuroendocrine tumors (TNETs) in most series, are very uncommon, and account for only two to five percent of all thymic cancers and less than 0.4% of all carcinoid tumors. These cancers are presumed to originate from the cells of the endocrine and nervous systems. They are more common in men with a male to female incidence ratio 3/1, with the peak incidence in the fifth decade of life. Patients may either be locally asymptomatic or present with cough, chest pain, or superior vena cave syndrome [2].

## Case presentation

**Figure 1:**
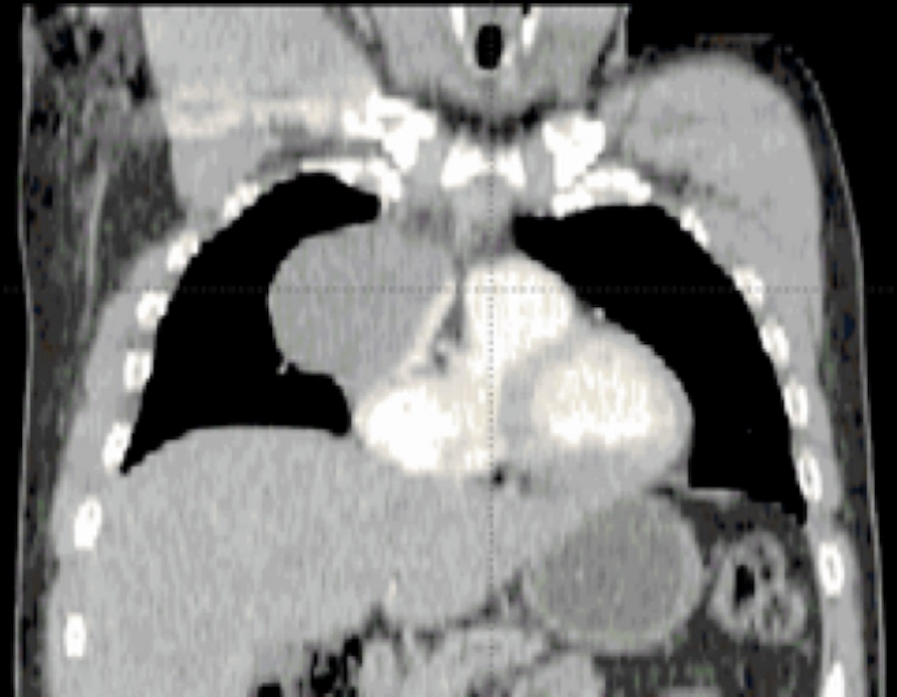
Coronal contrast-enhanced computed tomography scan of thorax showing the right anterior mediastinal mass.

**Figure 2:**
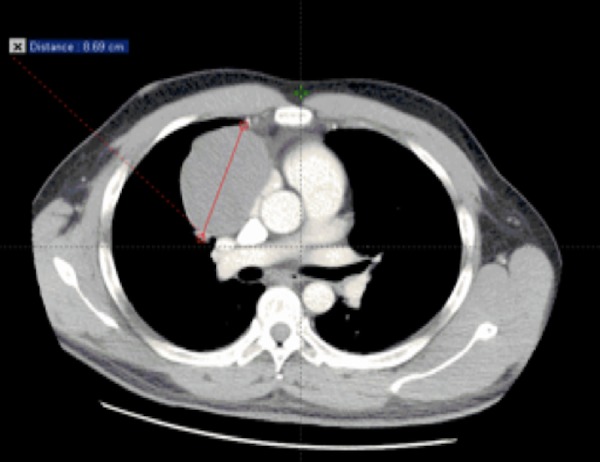
Axial contrast-enhanced computed tomography scan of thorax showing the right anterior mediastinal mass.

A 39-year-old man (former smoker) developed sudden and progressive pleuritic chest pain, dyspnea, and neck pain in November 2011. The patient rated this pain at 10 out of 10. A chest radiograph revealed an anterior mediastinal mass with a small right pleural effusion. A thoracic computed tomography (CT) scan showed a right anterior mediastinal mass of 6 × 8 × 7 cm (Figures 1-2). The mass appeared encapsulated. The mass compressed the inferior vena cava and was not associated with radiological adenopathy.

**Figure 3:**
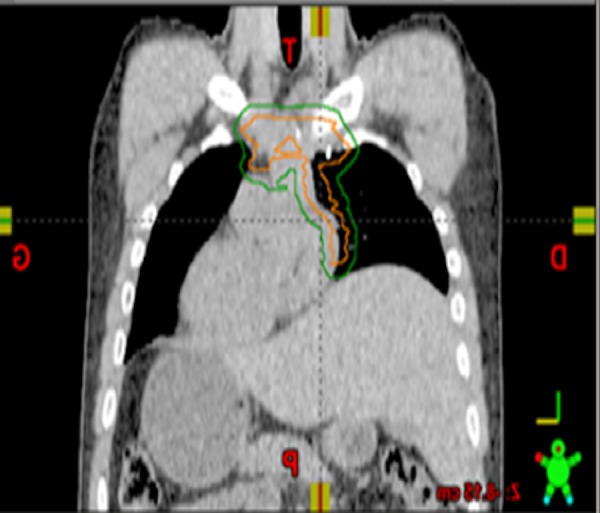
Coronal computed tomography image from the radiotherapy planning study with an outline of the clinical target volume (ORANGE) and the planning target volume (GREEN).

**Figure 4:**
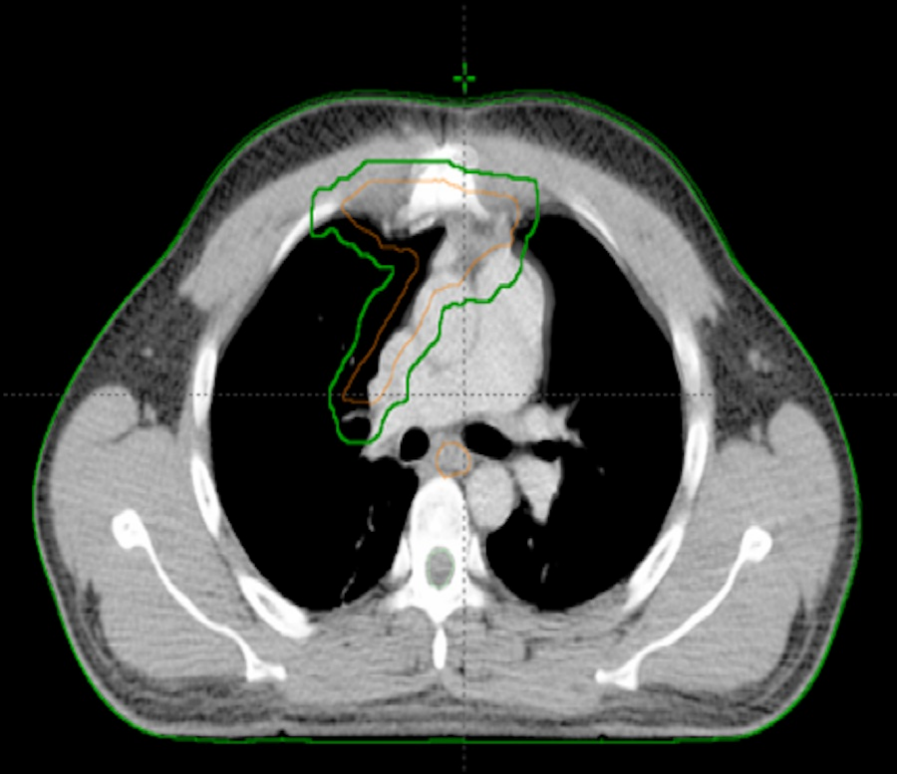
Axial computed tomography image from the radiotherapy planning study with an outline of the clinical target volume (ORANGE) and the planning target volume (GREEN).

The patient proceeded directly to the operating room without prior biopsy or neoadjuvant therapy. Through a median sternotomy, a radical en bloc resection was performed. The tumor was found in the anterior mediastinum with extension to the pericardia and right hilum. This required a radical thymectomy and a pericardectomy with a pericardial window. The resection has done and an iatrogenic defect in the left brachiocephalic vein required. No lymph node staging was performed. The surgeon defined the pericardial and hilar margins with clips to help guide potential post-operative radiotherapy. Pathology was that of an atypical thymic neuroendocrine carcinoid tumor. Macroscopically, the tumor was heterogeneous, encapsulated and yellowish in color, measured 10 × 10 × 6 cm. Microscopically, the tumor had central necrosis and two mitoses per 10 HPF. There was no perineural or vascular invasion. The tumor capsule was infiltrated but the surgical margin was negative. On immunohistochemistry cytokeratin AE1/AE3 was positive, cytokeratin 8/18 positive, chromogranin positive, CD-56 positive, and TTF1 negative. No c-KIT mutations were found. The tumor was staged as Masaoka IIA. The postoperative course was uneventful and the patient was released from the hospital after 11 days. Following surgery, the patient had noticeable improvement of his pain and dyspnea. A postoperative investigation was done to rule out metastases. A fluorodeoxyglucose positron emission tomography/computed tomography (FDG PET/CT) scan revealed no evidence of local, regional, or distant hypermetabolic activity. The Technetium-99m sestamibi (99m Tc-MIBI) (indium 111) scan was also negative for pathological uptake. The case was discussed in our multidisciplinary thoracic oncology tumor board and the recommendation was to offer adjuvant radiotherapy without chemotherapy. Preradiotherapy pulmonary function tests showed an FEV1 of 2.33 L (59%) pre-bronchodilation, and 2.53 L post-bronchodilation (64% of predicted). The diffusion capacity (DLCO) was 73% of predicted. A dose of 60 Gy over 30 daily fractions was selected in this postoperative adjuvant curative setting. In order to help define the clinical target volume, pre-operative imaging was co-registered to the planning 4D-CT. After breathing motion was taken into account to tumor bed, an additional 6 mm margin to the internal clinical target volume (ICTV) was used to create the investigator’s planning target volume (IPTV). The IPTV had a volume of 451 cm^3^ (Figures 3-4).

Our standard dose constraints for radiotherapy without concurrent chemotherapy were used in the planning process as shown in Table 1 [3].

**Table 1:** Dose constraints for thoracic radiation without concurrent chemotherapy.

Organ	Dose constraints
Spinal cord	Dmax < 45 Gy
Lung	MLD ≤ 20 Gy, V20 ≤ 30%
Heart	V30 ≤ 45%, Mean dose < 26 Gy
Esophagus	Dmax ≤ 66 Gy, Mean dose < 34 Gy
Kidney	20 Gy < 32% of bilateral kidney, V 50 < 18 Gy for each kidney
Liver	V50 < 30 Gy, V30 < 40%

**Figure 5:**
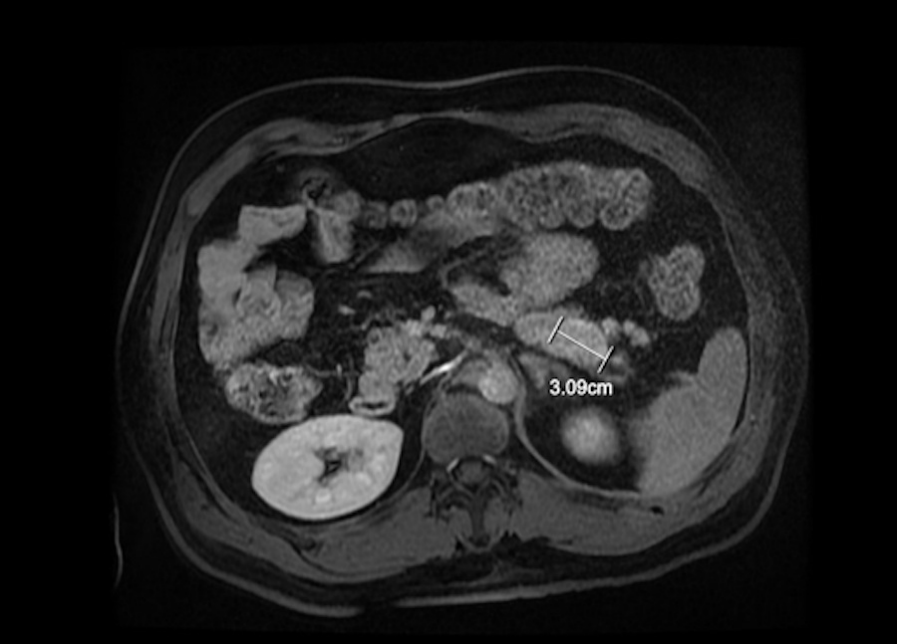
T1 axial post-gadolinium dynamic three-dimensional magnetic resonance imaging of abdomen showing an intrapancreatic lesion of 3 × 2 cm.

The treatment was delivered using helical tomotherapy with daily image guidance. The patient had grade 1 of common terminology criteria for adverse events (CTCAE) acute toxicity from radiotherapy: asthenia, odynophagia, dysphagia, cough, chest alopecia, and dermalgia. As the patient was found to have a family history of multiple endocrine neoplasia type 1 (MEN-1) a comprehensive workup was requested including prolactin, parathyroid hormone (PTH), catecholamine, chromogranin A, calcium, calcitonin, abdominal ultrasound, CT scan of abdomen, magnetic resonance imaging (MRI) of brain, gastrointestinal, and genetic evaluations. These investigations revealed the following levels: chromogranin A 667 ng/ml (<180 ng/ml), calcium 3.07 mmol/L (2.17-2.56 mmol/L), calcitonin 6 ng/L (<8.8 ng/L), and PTH 12.9 pmol/L (1.2-5.7 pmol/L). The patient was found to have a mutation of the Menin gene located on chromosome 11q13. He had a large deletion detectable by Southern blot analysis, confirming that the patient had the MEN 1 syndrome [2]. The brain MRI showed an 8 mm pituitary nodule, suggestive of a pituitary microadenoma. Subsequent parathyroid MIBI scintigraphy showed pathological uptake in the posterior pole of the inferior lobe of the right thyroid as well as suspicion of a second adenoma of the left lobe. The patient underwent a parathyroidectomy in the fall of 2012, revealing parathyroid hyperplasia. A follow-up somatostatin scan showed hyperactivity in the pancreas. The patient was further imaged with an abdominal MRI (Figure 5). This revealed a 3 × 2 cm intrapancreatic lesion for which he underwent a total pancreatectomy. Pathology of the pancreas showed three small low-grade well differentiated (Grade 1) neuroendocrine tumors of size 2.5, 0.4, and 0.8 cm, as well as one involved lymph node out of two (pT2 N1). Although pancreatic metastases from thymic neuroendocrine have been described, the pancreatic lesions were considered to be distinct neuroendocrine tumors (NETs).

The patient continues to have annual follow-up CT scans of the thorax. Now more than four years from his thoracic radiotherapy, the patient is clinically and radiologically free of recurrent disease.

## Discussion

According to the WHO 2004 classification, TNETs are included in the category of thymic carcinoma, (Type C) WHO classification [4], as shown in Table 2.

**Table 2:** WHO 2004 classification.

Type A:
Medullary thymoma
Spindle cell thymoma
Type AB:
Mixed thymoma
Type B1:
Lymphocyte rich
Predominantly cortical thymoma
Organoid thymoma
Lympochyte predominant thymoma
Lymphocytic thymoma
Type B2:
Cortical thymoma
Type B3:
Epithelial predominant thymoma
Squamoid thymoma
Well-differentiated thymic carcinoma
Type C:
Thymic carcinoma

Prior to 1972, various tumors of the thymus were grouped in the category of epithelial thymomas. In 1972, Rosai and Higa reported eight cases of thymic NETs and named them thymic carcinoids [5]. The TNETs are characterized as atypical on the basis of necrosis or local invasion. More than one grading systems for thymic carcinoids have been proposed (Table 3) [6].

**Table 3:** Grading of thymic neuroendocrine tumors.

Terminology	Rosai, et al.	Moran and Suster
Carcinoid	Carcinoid type (grade) 1	Well-differentiated neuroendocrine carcinoma (low grade)
Atypical carcinoid	Carcinoid type (grade) 2	Moderately-differentiated neuroendocrine carcinoma (intermediate grade)
Small cell lung cancer	Carcinoid type (grade) 3	Poorly-differentiated neuroendocrine carcinoma (high grade)

According to Filoso, typical carcinoid represents 28%, atypical carcinoid 40%, and poorly differentiated 28% of TNET cases. There was no data on type for 13% [2]. Immunohistochemical markers help to specify the type of thymic tumor as shown in Table 4 [7].

**Table 4:** Immunohistochemical markers.

	Epithelial markers	Miscellaneous markers of thymic carcinoma	Neuroendocrine markers	Lymphoid markers of mature T phenotype	Lymphoid markers of immature T phenotype	Lymphoid markers: CD 20	
	Cyto-keratine	CD117, CD5, CD70, EMA	Synaptophysin, chromogranin, CD56	CD3, CD45	CD99, Tdt, CD1a	LY	EC
Thymoma	+	-	-	+	+	-	-/+
Thymic hyperplasia	+	-	-	+	+	+	-
Thymic carcinoma	+	+	+/-	+	-	-	-
Thymic neuroendocrine tumors	+	-	+	-	-	-	-

Almost 50% of thymic neuroendocrine carcinomas are associated clinically with endocrine abnormalities because of an association with other endocrine tumors, the MEN type 1 syndrome or Cushing syndrome. Autoimmune disease is rarely associated with thymic carcinoid [8]. Table 5 describes paraneoplastic syndromes associated with thymic carcinoid [8].

**Table 5:** Paraneoplastic syndromes associated with thymic carcinoid.

Syndrome	Thymoma	Thymic carcinoma	Thymic carcinoid
None	2623 (61%)	607 (95%)	119 (96%)
Myasthenia gravis	1634 (38%)	31 (5%)	5 (4%)
Hypogammaglobulinemia	13 (<1%)	1 (<1%)	0 (0%)
Red cell aplasia	37 (1%)	1 (<1%)	0 (0%)
Unknown	611 (14%)	208 (33%)	36 (29%)

They are most often staged using the Masaoka or Masaoka-Koga staging system for thymoma as shown in Table 6 [9].

**Table 6:** Masaoka and Masaoka-Koga staging of thymoma.

Masaoka	Masaoka-Koga
I: Macroscopically and microscopically completely encapsulated and microscopically no capsular invasion	I: Grossly and microscopically completely encapsulated tumor
II: Invasion beyond the capsule and into nearby fatty tissue or to the pleura	
IIA: Microscopic invasion of capsule	IIA: Microscopic *transcapsular* invasion
IIB: Macroscopic invasion into surrounding fatty tissue or mediastinal pleura	IIB: Macroscopic invasion into thymic or surrounding fatty tissue, or grossly adherent to but not breaking through mediastinal pleura or pericardium
III: Macroscopic invasion into neighboring organs (i.e., pericardium, great vessels, or lung)	III: Macroscopic invasion into neighboring organ (i.e., pericardium, great vessel, or lung)
IVA: Pleural or pericardial dissemination	IVA: Pleural or pericardial metastases
IVB: Lymphogenous or hematogenous metastasis	IVB: Lymphogenous or hematogenous metastasis

Although there are some differences in definition for stage I and II, the survival of these two stages is very similar and this does not interfere with interpretation of data from studies. Seventy percent cases of thymic carcinoid present with stage III-IV [2]. In thymic tumor at large, without segregation by tumor type of treatment intent, 2/3 of deaths are tumor related. In thymic NET, death is mostly commonly tumor related.

Masaoka stage is a significant predictor of survival in TNET. Tiffet reported on 12 patients: the median survival time was 6.8 years for stage I, 6.3 years for stage II, six years for stage III, 2.25 years for patients with stage IVA disease, and 3.3 years for stage IVB [9]. According to International Thymic Malignancy Interest Group (ITMIG), the median survival is 7.5 years for all stages. In 148 cases, the median survival is 13.5 years for stage I-II, 7.3 years for stage III, 3.8 years for stage IVa, and 4.2 years for stage IVb [1]. Radical resection with lymph node dissection (when feasible) remains the standard treatment, and because of proximity from the pericardia or phrenic nerve, it frequently will lead to positive margins. Microscopic residual disease will often lead to local recurrence. Completeness of resection predicts for recurrence and death, but there may be little difference in the extent of residual disease R1 vs R2. Histologic grading of NETs may not be prognostic [2].

The role of adjuvant radiation in thymic carcinoid is still unclear. Postoperative radiotherapy has been recommended to increase local control (especially if the resection was incomplete), and preoperative radiation has been suggested to facilitate surgery. In either case, radiation can be intended to delay or avoid distant metastases. Almost half of the thymic NETs are characterized as aggressive tumors and there is a high risk of metastases, with a tendency to invade local structures and metastasize outside the thorax in 20-30%. The most common sites of metastases are lungs and pleura, bones, liver, spleen, brain, and adrenal glands. It is hard to find data on the site of first recurrence, local or at distance. Most of our knowledge on treatment of thymic neuroendocrine tumors comes from case reports or small case series [8].

The ITMIG, and the Surveillance, Epidemiology, and End Results (SEER) databases include the largest numbers of thymic tumors [1]. Filosso, et al. [2] reported on a large series from the ITMIG and the European Society of Thoracic Surgeons database for primary outcomes of neuroendocrine tumors of the thymus. From 1984 to 2012, 205 patients were surgically resected. Radiotherapy was applied in 81 patients (40%), mostly in the adjuvant setting. Radiotherapy was administered after surgery in 70 patients, prior to surgery in six and with palliative intent in five. Radiotherapy was given in the adjuvant setting mainly due to local invasiveness, incompleteness of resection, or lymph node involvement. In this large retrospective series, radiotherapy was not significantly associated with survival outcomes [2]. In none of the series were risk factors accounted for in evaluating the impact of radiotherapy.

Tiffet, et al. reported 12 cases of thymic NET. He found that the adjuvant radiotherapy increased local control. Nine of 12 patients had distant metastases (82%). Nine patients had R0 disease; in these nine patients five of them did not receive radiotherapy and four had local recurrences, one patient died one month after the surgery and three patients had radiotherapy and they did not have local recurrences. Three patients had R1-R2 disease and all had local recurrences even if two of them had chemoradiation post-op [10].

Gaur, et al. published a description of the treatments received as derived from SEER database. From 1973 to 2006, out of the 156 patients where radiation therapy data were available, 70 had received radiation as part of their primary therapy. Eleven percent had initially localized disease, 56% regional, and 58% metastatic. Radiation was more commonly administered to patients with more advanced stage. No significant survival benefit could be shown for radiation — but these patients were likely pre-selected for poor prognostic factors [1].

In Table 7 we did a summary of all studies that were published about Thymic neuroendocrine.

**Table 7:** Summary of publications about Thymic neuroendocrine.

	Year	Number of patients	Adjuvant radiotherapy
Moran, et al.	1997	80	No information
Gaur, et al.	2010	160	43.7%
Filosso, et al.	2015	205	39.5%
Tiffet, et al.	2003	12	50.0%
Villa, et al.	1994	14	No information

## Conclusions

Thymic neuroendocrine tumors are aggressive tumors for which survival is influenced mainly by stage and completeness of resection. Because of their rarity, a clear role for radiation is not well established. We favor radiation in adjuvant setting for most patients. Our patient, stage II R0, was offered radiation because of the aggressivity of his tumor. Our patient received modern radiation treatments with low toxicity and has been free of recurrence for more than five years.
